# Breast cancer literacy among higher secondary students: results from a cross-sectional study in Western Nepal

**DOI:** 10.1186/s12885-016-2166-8

**Published:** 2016-02-18

**Authors:** Parash Mani Bhandari, Kiran Thapa, Sarmila Dhakal, Shristi Bhochhibhoya, Rashmi Deuja, Pawan Acharya, Shiva Raj Mishra

**Affiliations:** Maharajgunj Medical Campus, Institute of Medicine, Tribhuvan University, Kathmandu, Nepal; Unit of Health Promotion Research, University of Southern Denmark, Esbjerg, Denmark; Nepal Development Society (NEDS), Chitwan, Nepal; School of Population Health, University of Western Australia, Crawley, WA Australia

**Keywords:** Breast cancer, Cross-sectional studies, Risk factors, Knowledge, Students, Nepal

## Abstract

**Background:**

Being the most common cancer among women worldwide, it is vital to be well-aware of breast cancer risk factors, symptoms and curability. However, few studies have reported breast cancer literacy in students using a validated instrument.

**Methods:**

A cross-sectional study was conducted among students of grades 11 and 12 from eleven higher secondary schools, one selected randomly from each *ilaka* of Parbat district. Questionnaire with modified Comprehensive Breast Cancer Knowledge Test was self-administered to 516 students. Knowledge score was categorized into two categories: ‘good knowledge’ and ‘poor knowledge’ taking median score as the cut-off. Chi-square test was used to determine difference in knowledge by socio-demographic factors, including gender.

**Results:**

Only 4.8 % of the students responded correctly to at least half of the items, and 1.4 % did not respond correctly to any of the items on risk factors and curability. Physical exercise was identified as a protective factor of breast cancer by 62.4 % of the students. Presence of noncancerous breast lumps (56.6 %) and being overweight (36.4 %) were recognized as the risk factors. Knowledge of lumpectomy and radiation therapy for treatment of breast cancer was reported by 42.8 % of students, while only 39.0 % were aware of the availability of treatment therapies other than mastectomy. Males were significantly better informed than females (*χ*^2^ = 4.02, *p* = 0.045). Pain in the breast (23.3 %), change in the shape of the breast (20.0 %) and discharge of pus (14.1 %) were the three most commonly recognized symptoms. Nearly one in two (47.1 %) students indicated that the school curriculum inadequately informed them on breast cancer.

**Conclusion:**

Our study demonstrates poor knowledge on breast cancer risk factors, symptoms and curability among higher secondary school students in Western Nepal. Still, several myths regarding breast cancer persist. Half of the students had the perception that school curriculum inadequately informed them on breast cancer. Future studies should aim at the measures necessary to address the inadequate knowledge, along with the perceived gap in school curriculum.

**Electronic supplementary material:**

The online version of this article (doi:10.1186/s12885-016-2166-8) contains supplementary material, which is available to authorized users.

## Background

Global statistics suggest that breast cancer is the most frequently diagnosed and a leading cause of death in women [1]. In South Asia, it is the most common malignancy among women [2], is detected more often in younger females and at a more advanced stage as compared to females of other regions [1]. This is partly due to inaccessibility to screening and diagnostic facilities, illiteracy, lack of prompt decision making and females being uninformed about early detection of breast cancer [3]. Preparedness and regular monitoring of cancer is the weakest in this region as population-based registries, plans of action and strategies are almost non-existent, which further adds to the agony. Targeting the modifiable factors like illiteracy regarding breast cancer symptoms and its early detection ameliorates the disease burden at the population level. With the refutation of the long-held belief that South Asian population are at a lower risk of breast cancer than the rest of the world [4], studies assessing the level of awareness and the state of preparedness are deemed further essential.

A bitter truth -- Nepal has no national registries for cancer and the reported prevalence of cancer varies with different studies [5]. A hospital based study suggests that knowledge regarding breast cancer is low [6], despite it being the second most common cancer among women in Nepal [7]. The incidence of breast cancer is high among younger, premenopausal women; most of the cases being diagnosed at an advanced stage -- when the likelihood of successful treatment is very low [8, 9]. Clinical evidence reveals that breast cancer at a younger age has a greater likelihood of death owing to delayed diagnosis of the disease [10]. Therefore, higher rates of breast cancer in younger age demand early intervention.

Interventions promoting healthy behaviors and practices in early adolescence have a great role in preventing breast cancer as some of these behaviors are developed at this stage of life. Targeting adolescents is crucial for the success of any prevention efforts, also because they constitute a major proportion of population. Adolescent students are very receptive to information [11], and therefore, healthy behaviors established at this phase of life are most likely to be continued [12]. Similarly, the role of men in health service provision and decision making is well noted [13]. However, very few studies have considered assessing men’s knowledge and awareness in relation to breast cancer [13–15].

In light of the evidences mentioned above, this study aims to explore the literacy of breast cancer risk factors, curability and its symptoms among the higher secondary students of Western Nepal. We focus on the difference in knowledge by socio-demographic factors, including gender.

## Methods

### Study design and setting

A cross-sectional study among the higher secondary students of Parbat district of Nepal was conducted. Parbat is a hilly district in Western Nepal with an area of 494 km^2^, population of 146,590 in 2011 of which 29,312 are aged 14–22 years and 12,645 attend a school [16]. Districts in Nepal have lower administrative divisions known as *ilakas* (sub-districts) and Parbat district consists of 11 such *ilakas*. Parbat represents a typical Nepalese hilly community with shades of both the urban and the rural lifestyles.

### Sample size and selection of participants

**Fig. 1:**
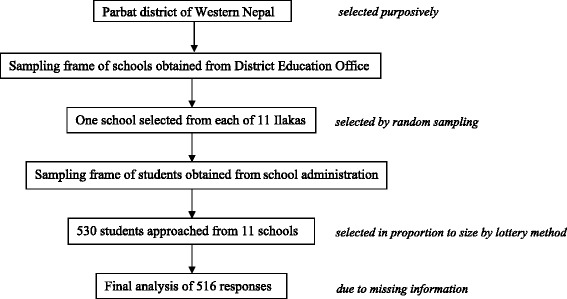
Steps of sample recruitment

Considering that 25 % of students are acquainted to the risk factors and symptoms of breast cancer [17], a sample size of 520 was optimal for a desired 95 % confidence interval, considering 4 % allowable error and 15 % non-response rate. The process of sampling involved random selection of a school from each *ilaka*. In the second stage, the principals of respective schools were contacted, briefed about the study objectives and asked for their permission to collect data. In order to attain the desired sample size, the number of students to be recruited from each school in proportion to the enrollment size was determined. Lottery method was used to randomly select the allocated number of students from attendance register at each school. Out of 530 students approached, none declined from participation in the study. Complete information was obtained from 516 participants and the responses of 14 participants were discarded because of missing information (Fig. 1).

### Data collection

The study objectives along with the correct technique to provide responses were explained and the questionnaires were distributed to the students. PMB, KT, SD, SB and RD carried out the data collection at the regular classroom of the students. Teachers were not present in the classroom at the time of enumeration, and the students were seated such that anonymity of responses was assured.

The Comprehensive Breast Cancer Knowledge Test (CBCKT) was used, with some contextual modifications. The original tool consists 20 items (12 statements on general awareness and 8 statements on curability), with reliability coefficient of 0.71 [18]. Two statements from the original tool were removed as they were specific to women of United States. An addition of 5 statements was made to the questionnaire considering the recently available evidences on the effects of early menarche, delayed menopause, physical activity, oral contraceptive pills and breastfeeding on breast cancer [19, 20]. Response alternatives included ‘Yes’, ‘No’ and ‘Don’t know’ (see Additional file 1). The questionnaire, which was in Nepali language, was previously pretested to a sample of 40 students from a higher secondary school of Kathmandu district, and was reviewed for language and comprehension.

Demographic information (age, sex, ethnicity and religion) and status of tobacco and alcohol consumption were obtained from the participants. Information regarding the family structure, family history of cancer and monthly family income of the students were also recorded. Students were asked for their perspectives on the adequacy of the breast cancer information in their curriculum. Sources of information regarding breast cancer and breast cancer symptoms, that were originally not a part of the CBCKT, were also recorded. Students were provided with a list of symptoms as options, and were asked to select potential symptoms of breast cancer.

### Data analysis

Data entry was carried out in EpiData 3.1 while all the analyses were performed in SPSS version 21 (SPSS, Chicago, Illinois, USA). Frequency and percentage were generated for responses to knowledge statements and socio-demographic variables. For a correct response to any of the 23 items on the questionnaire, a score of 1 was provided while an incorrect response or a response indicating ‘Don’t know’ was scored 0. A composite score of all the items combined was calculated which could range from 0–23. Median value of the composite score was 7.0 (slightly greater than the mean: 6.65). Knowledge score, after dichotomization taking the median as the cut-off, was grouped into two categories: ‘good knowledge’ and ‘poor knowledge’. Comparison of knowledge level with personal characteristics of students was made using chi-squared test, *p*-value <0.05 taken as statistically significant.

### Ethics

This study was approved by the Department of Community Medicine and Public Health (DCMPH), Maharajgunj Medical Campus (Ref. No. 105-072-073). During the ethical review, the need for parental consent for participants aged <16 years was waived by the DCMPH owing to the non-interventional and non-invasive study design. Prior to data collection, permission was obtained from the District Education Office and school principals. We obtained written informed consent from all the participants after explaining the study objectives. No financial incentive was provided to the participants. Participants were assured of anonymity and were informed of their right to voluntary participation and withdraw from the study at any stage.

## Results

### Characteristics of participants

Participants belonged to an age group of 14 to 22 years with mean of 16.92 years (SD = 1.13). More than half of the participants (54.8 %) were female, 71.3 % were Brahmin/Chhetri and 95.0 % were Hindu. A majority of the participants (62.0 %) studied in grade 12, 1.9 % were tobacco users and 1.7 % consumed alcohol. One in every five participants (20.2 %) had a family history of cancer. Knowledge of students varied significantly with gender (*χ*^2^ = 4.02, *p* = 0.045) (Table 1).

**Table 1:** Characteristics of participants and their knowledge level

Characteristics	Total	Poor knowledge^a^	Good knowledge^a^	*p*-value^b^
Age (years)				
Mean ± SD = 16.92 ± 1.13 years				
14-16 years	185 (35.9)	93 (50.3)	92 (49.7)	0.849
17-19 years	321 (62.2)	168 (52.3)	153 (47.7)	
20-22 years	10 (1.9)	5 (50.0)	5 (50.0)	
Sex				
Male	233 (45.2)	102 (43.8)	131 (56.2)	0.045*
Female	283 (54.8)	149 (52.7)	134 (47.3)	
Ethnicity				
Brahmin/Chhetri	368 (71.3)	185 (50.3)	183 (49.7)	0.440
Aadiwasi/Janajati	65 (12.6)	26 (40.0)	39 (60.0)	
Dalit	47 (9.1)	24 (51.1)	23 (48.9)	
Others	36 (7.0)	16 (44.4)	20 (55.6)	
Religion				
Hindu	490 (95.0)	239 (48.8)	251 (51.2)	0.794
Others	26 (5.0)	12 (46.2)	14 (53.8)	
Grade				
11	196 (38.0)	94 (48.0)	102 (52.0)	0.808
12	320 (62.0)	157 (49.1)	163 (50.9)	
Tobacco use				
Yes	10 (1.9)	7 (70.0)	3 (30.0)	0.172
No	506 (98.1)	244 (48.2)	262 (51.8)	
Alcohol consumption				
Yes	9 (1.7)	5 (55.6)	4 (44.4)	0.676
No	507 (98.3)	246 (48.5)	261 (51.5)	
Type of family				
Nuclear family	378 (73.3)	183 (48.4)	195 (51.6)	0.862
Extended family	138 (26.7)	68 (49.3)	70 (50.7)	
Monthly family income				
≤NPR 20,000 (≤20 USD)	358 (69.4)	176 (49.2)	182 (50.8)	0.723
>NPR 20,000 (>20 USD)	158 (30.6)	75 (47.5)	83 (52.5)	
Family history of cancer				
Present	104 (20.2)	42 (40.4)	62 (59.6)	0.059
Absent	412 (79.8)	209 (50.7)	203 (49.3)	

Data are frequency (percentage)

^a^Row percentage

^b^ *p*-value derived from the chi-square test of association

*p <0.05

Nearly half of the participants (47.1 %) perceived that the school curriculum inadequately informed them on breast cancer. One-in-four students (24.8 %) had heard of Breast Self Examination (BSE) while 29.8 % had heard of mammography. Only 4.8 % of the students responded correctly to at least half of the 23 items, and 1.4 % did not respond correctly to any of the items on risk factors and curability.

### Risk factors of breast cancer

Breast cancer was correctly identified as the most common cancer in women by 49.0 % (male: 48.4 %; female: 49.5 %) of the students. Regarding the knowledge of risk factors of breast cancer, most of them (49.4 %- male: 52.8 %; female: 46.6 %) were aware of the protective role of breastfeeding and exercise (62.4 %- male: 66.1 %; female: 59.4 %). Respondents correctly reported risk factors of breast cancer like early menarche (19.2 %- male: 25.3 %; female: 14.1 %), delayed menopause (28.3 %- male: 36.1 %; female: 21.9 %), use of oral contraceptives (35.7 %- male: 36.1 %; female: 35.3 %) and being overweight (36.4 %- male: 43.3 %; female: 30.7 %). Similarly, 25.4 % (male: 29.6 %; female: 21.9 %) of the students incorrectly reported childbearing before age of 30 to be a risk factor for breast cancer. Further, 22.3 % (male: 22.3 %; female: 22.3 %) of them were aware that risk of breast cancer gets higher in older age. Only 11.6 % (male: 9.9 %; female: 13.1 %) of the students correctly responded that a woman without any known risk factor may also develop breast cancer later in her life. Increased risk of breast cancer with the occurrence of some fibrocystic breast disease was correctly reported by 56.6 % (male: 52.8 %; female: 59.7 %) of the students.

Students had false perception that hard blow to breast (35.1 %- male: 34.8 %; female: 35.3 %) and irritation caused by a brassiere (25.0 %- male: 21.0 %; female: 28.3 %) triggers breast cancer. Similarly, 42.2 % (male: 41.2 %; female: 43.1 %) wrongly responded that most of the lumps in breast are cancerous. Also, 30.8 % (male: 29.2 %; female: 32.2 %) were misinformed that breast cancer is rare in older women (aged >70 years) (Table 2).

**Table 2:** Response of participants to statements on risk factors of breast cancer

Statement	Response	Total (*n* = 516)	Male (*n* = 233)	Female (*n* = 283)	*p*-value^c^
Early menarche in a woman increases the risk of developing breast cancer.^a^	Yes	99 (19.2)	59 (25.3)	40 (14.1)	0.006*
	No	106 (20.5)	44 (18.9)	62 (21.9)	
	Don’t know	311 (60.3)	130 (55.8)	181 (64.0)	
Women who have delayed menopause are at greater risk of breast cancer. ^a^	Yes	146 (28.3)	84 (36.1)	62 (21.9)	0.002**
	No	62 (12.0)	24 (10.3)	38 (13.4)	
	Don’t know	308 (59.7)	125 (53.6)	183 (64.7)	
Use of oral contraceptives increases a woman’s risk of developing breast cancer.^a^	Yes	184 (35.7)	84 (36.1)	100 (35.3)	0.876
	No	63 (12.2)	30 (12.9)	33 (11.7)	
	Don’t know	269 (52.1)	119 (51.0)	150 (53.0)	
Breastfeeding reduces the risk of developing breast cancer.^a^	Yes	255 (49.4)	123 (52.8)	132 (46.6)	0.381
	No	69 (13.4)	29 (12.4)	40 (14.1)	
	Don’t know	192 (37.2)	81 (34.8)	111 (39.3)	
Physical exercise reduces the risk of developing breast cancer.^a^	Yes	322 (62.4)	154 (66.1)	168 (59.4)	0.287
	No	43 (8.3)	18 (7.7)	25 (8.8)	
	Don’t know	151 (29.3)	61 (26.2)	90 (31.8)	
A hard blow to the breast may cause a woman to get breast cancer later in life.^b^	Yes	181 (35.1)	81 (34.8)	100 (35.3)	0.565
	No	77 (14.9)	39 (16.7)	38 (13.4)	
	Don’t know	258 (50.0)	113 (48.5)	145 (51.3)	
The constant irritation of a tight bra can, over time, cause breast cancer.^b^	Yes	129 (25.0)	49 (21.0)	80 (28.3)	0.121
	No	89 (17.2)	39 (16.7)	50 (17.7)	
	Don’t know	298 (57.8)	145 (62.3)	153 (54.0)	
In some women, being overweight increases the risk of developing breast cancer.^a^	Yes	188 (36.4)	101 (43.3)	87 (30.7)	0.011*
	No	86 (16.7)	33 (14.2)	53 (18.7)	
	Don’t know	242 (46.9)	99 (42.5)	143 (50.6)	
A woman who bears her first child before the age of 30 is more likely to develop breast cancer than a woman who bears her first child after the age of 30.^b^	Yes	131 (25.4)	69 (29.6)	62 (21.9)	0.067
	No	282 (54.7)	49 (21.0)	54 (19.1)	
	Don’t know	103 (19.9)	115 (49.4)	167 (59.0)	
Women with no known risk factors for breast cancer rarely get breast cancer.^b^	Yes	167 (32.4)	84 (36.1)	83 (29.3)	0.202
	No	60 (11.6)	23 (9.9)	37 (13.1)	
	Don’t know	289 (56.0)	126 (54.0)	163 (57.6)	
Some types of fibrocystic breast disease (noncancerous breast lumps) increase a woman’s risk of breast cancer.^a^	Yes	292 (56.6)	123 (52.8)	169 (59.7)	0.074
	No	32 (6.2)	20 (8.6)	12 (4.2)	
	Don’t know	192 (37.2)	90 (38.6)	102 (36.1)	
Breast cancer is more common in 65-year-old women than in 40-year-old women.^a^	Yes	115 (22.3)	52 (22.3)	63 (22.3)	0.054
	No	160 (31.0)	84 (36.1)	76 (26.9)	
	Don’t know	241 (46.7)	97 (41.6)	144 (50.8)	
The most frequently occurring cancer in women is breast cancer.^a^	Yes	253 (49.0)	113 (48.4)	140 (49.5)	0.018*
	No	107 (20.7)	60 (25.8)	47 (16.6)	
	Don’t know	156 (30.3)	60 (25.8)	96 (33.9)	
Women over age 70 rarely get breast cancer.^b^	Yes	159 (30.8)	68 (29.2)	91 (32.2)	0.434
	No	88 (17.1)	45 (19.3)	43 (15.2)	
	Don’t know	269 (52.1)	120 (51.5)	149 (52.6)	
Most breast lumps are cancerous.^b^	Yes	218 (42.2)	96 (41.2)	122 (43.1)	0.064
	No	87 (16.9)	49 (21.0)	38 (13.4)	
	Don’t know	211 (40.9)	88 (37.8)	123 (43.5)	

Data are frequency (percentage)

^a^Statement is true

^b^Statement is false

^c^ *p*-value derived from the chi-square test of association

^*^ *p* <0.05, ^**^ *p* < 0.005

### Curability of breast cancer

About two in every five (39.0 %- male: 41.6 %; female: 36.7 %) respondents were informed that modalities of treatment other than mastectomy are also available and 42.8 % (male: 38.2 %; female: 46.6 %) knew that lumpectomy and radiation therapy are available alternatives for treatment of breast cancer. Less than half (48.3 %- male: 52.4 %; female: 44.9 %) of the students held a misperception that successful treatment of cancerous breast lump after it becomes painful is next to impossible while 21.1 % (male: 25.3 %; female: 17.7 %) believed that treatment of breast cancer without removal of all the lymph glands around breast and under the arm is not possible.

A significant proportion (45.9 %- male: 48.5 %; female: 43.8 %) of the students believed that effective treatment of breast cancer after it can be felt by a woman is not possible, and 39.5 % (male: 41.6 %; female: 37.8 %) had the belief that the chances of successful treatment is better upon removal of the whole breast. Similarly, 29.3 % (male: 33.5 %; female: 25.8 %) of the participants believed that having a family history of breast cancer decreased the likelihood of breast cancer being cured. Also, 35.9 % (male: 31.3 %; female: 39.6 %) thought that despite the early detection and treatment, a woman with breast cancer cannot live a normal life (Table 3).

**Table 3:** Response of participants to statements on curability of breast cancer

Statement	Response	Total (*n* = 516)	Male (*n* = 233)	Female (*n* = 283)	*p*-value^c^
For many women, breast cancer can now be successfully treated without breast removal (mastectomy).^a^	Yes	201 (39.0)	97 (41.6)	104 (36.7)	0.035*
	No	94 (18.2)	50 (21.5)	44 (15.5)	
	Don’t know	221 (42.8)	86 (36.9)	135 (47.8)	
By the time a cancerous breast lump is painful, it is too late to be successfully treated.^b^	Yes	249 (48.3)	122 (52.4)	127 (44.9)	0.142
	No	66 (12.8)	31 (13.3)	35 (12.4)	
	Don’t know	201 (38.9)	80 (34.3)	121 (42.7)	
If all lymph glands around the breast and under the arm are not removed, breast cancer cannot be cured.^b^	Yes	109 (21.1)	59 (25.3)	50 (17.7)	0.098
	No	76 (14.7)	31 (13.3)	45 (15.9)	
	Don’t know	331 (64.2)	143 (61.4)	188 (66.4)	
Breast cancer is sometimes treated successfully by removal of the lump (lumpectomy) and radiation therapy.^a^	Yes	221 (42.8)	89 (38.2)	132 (46.6)	0.100
	No	57 (11.0)	31 (13.3)	26 (9.2)	
	Don’t know	238 (46.2)	113 (48.5)	125 (44.2)	
Breast cancer is less likely to be cured in women with a family history of breast cancer than in women with no family history of breast cancer.^b^	Yes	151 (29.3)	78 (33.5)	73 (25.8)	0.155
	No	124 (24.0)	54 (23.2)	70 (24.7)	
	Don’t know	241 (46.7)	101 (43.3)	140 (49.5)	
By the time a woman can feel a cancerous breast lump, it is too late to treat it effectively.^b^	Yes	237 (45.9)	113 (48.5)	124 (43.8)	0.132
	No	87 (16.9)	44 (18.9)	43 (15.2)	
	Don’t know	192 (37.2)	76 (32.6)	116 (41.0)	
Even if breast cancer is caught very early, the chances for cure are much better if the whole breast is removed.^b^	Yes	204 (39.5)	97 (41.6)	107 (37.8)	0.053
	No	96 (18.6)	51 (21.9)	45 (15.9)	
	Don’t know	216 (41.9)	85 (36.5)	131 (46.3)	
Even if detected and treated early, a woman with breast cancer is unlikely to live a normal life span.^b^	Yes	185 (35.9)	73 (31.3)	112 (39.6)	0.118
	No	203 (39.3)	95 (40.8)	108 (38.2)	
	Don’t know	128 (24.8)	65 (27.9)	63 (22.2)	

Data are frequency (percentage)

^a^Statement is true

^b^Statement is false

^c^ *p*-value derived from the chi-square test of association

*p <0.05

### Symptoms of breast cancer

Only 39.5 % of the students chose at least one symptom of breast cancer from the list provided. Pain in the breast was the most commonly suggested symptom, reported by 23.6 % of male students and 23.0 % of female students. Change in shape of the breast was the second most common symptom reported; 20.6 % of males and 19.4 % of females mentioned it (Table 4).

**Table 4:** Knowledge of breast cancer symptoms

Symptoms^a^	Total (*n* = 516)		Male (*n* = 233)		Female (*n* = 283)	
	Yes	No	Yes	No	Yes	No
Pain in breast	120 (23.3)	396 (76.7)	55 (23.6)	178 (76.4)	65 (23.0)	218 (77.0)
Change in breast shape	103 (20.0)	413 (80.0)	48 (20.6)	185 (79.4)	55 (19.4)	228 (80.6)
Discharge of pus	73 (14.1)	443 (85.9)	36 (15.5)	197 (84.5)	37 (13.1)	246 (86.9)
Painless lump in breast	59 (11.4)	457 (88.6)	25 (10.7)	208 (89.3)	34 (12.0)	249 (88.0)
Increase in the number of breast lumps	44 (8.5)	472 (91.5)	20 (8.6)	213 (91.4)	24 (8.5)	259 (91.5)
Weight loss	43 (8.3)	473 (91.7)	26 (11.2)	207 (88.8)	17 (6.0)	266 (94.0)
Nausea	18 (3.5)	498 (96.5)	11 (4.7)	222 (95.3)	7 (2.5)	276 (97.5)

^a^Multiple-response

Data are frequency (percentage)

### Source of information regarding breast cancer

**Fig. 2:**
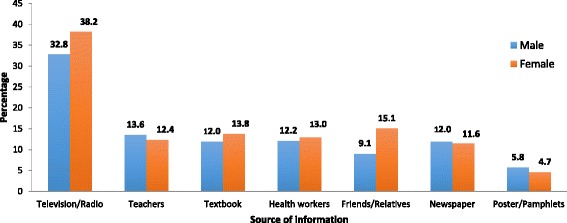
Sources of information

The majority of students (70.9 %) reported television/radio as the most common source of information on breast cancer. Other sources, reported in descending order of frequency were: teachers (26.0 %), textbooks (25.8 %), health workers (25.2 %), friends/relatives (24.2 %), newspapers (23.6 %) and poster/pamphlets (10.5 %) (Fig. 2).

## Discussion

Most of the students had misleading information about age, early menarche and delayed menopause as the risk factors. More than half of the respondents, regardless of their sex, were not well-informed about the risk possessed by regular intake of oral contraceptive pills (OCPs).

Many respondents did not know that breast cancer can be prevented at an early stage if diagnosed properly. A substantial proportion of students had myths that early detection of breast cancer has no benefits on success of treatment, and hard blow to the breast causes breast cancer. This depicts the abysmal level of awareness and ignorance of risk factors in the community. Being less aware about the screening methods and treatment modalities can be a barrier to seek screening and detection services thus causing a delay in diagnosis and treatment. In breast cancer, the more advanced the disease, the lower the chance of survival [21].

Age, educational level, household income and family history of breast cancer are reported to be significantly associated with the knowledge level [22, 23]. However, the study did not reveal such association. The study shows that male students were better informed of breast cancer risk factors and curability than their female counterparts. This finding is consistent with the findings of a previous study by McMenamin et al. [15]. Though females are at a higher risk of the disease, they are generally less informed than the male population. This apparent difference in knowledge between the genders might be accounted for difference in access to health information between genders, and social restrictiveness.

By knowing a simple measure like BSE, women can decrease the risk of breast cancer by early detection and prompt treatment. The study showed that less than one-fourth of the respondents had heard of BSE. Of the people who have heard of BSE, few know the correct practice [24, 25]. Practice of BSE, as explained by the Health Belief Model, is shaped by the interaction of perceived susceptibility, benefits, perceived seriousness, self-efficacy and perceived barriers [25, 26]. So, with fewer percentage of respondents who have merely heard of BSE, one can easily imagine that, in this study, students who can actually perform it might be negligible.

The most common source of information related to the breast cancer was Television/Radio which is similar to the findings reported by previous studies [27, 28]. This stresses that health education programs through Television/Radio can be most effective in promoting and modifying healthy behavior in our study population. Teachers, Textbook, Health workers and Friends/relatives share similar but account for low percentage in the source of information.

Knowledge regarding the symptoms of breast cancer is of importance as a layman seeks health services depending on his/her literacy of these symptoms. In this study, only less than half of the students could mention at least one symptom of breast cancer. The three most commonly cited symptoms of breast cancer were pain in breast, change in breast shape and discharge of pus from the nipple. Our findings are consistent with a previous study conducted among Australian women, in which these three symptoms were reported by a major proportion of participants [29].

It is important to note that in most of the items, a high percentage of the students replied with a ‘Don’t know’ response. This depicts that the students have no idea regarding the risk factors and curability of breast cancer. Further, nearly half of the students perceived existing school curriculum inadequate to be well-aware on breast cancer.

As higher secondary students are in age of making reproductive choices; they should, therefore, be aware about the risks linked with oral contraceptive use. While most of the biological risk factors are non-modifiable, some behaviors like use of oral contraceptive, physical exercise, breastfeeding and initiation of regular BSE at an early age can reduce the risk of developing breast cancer. There is a scope to promote BSE among the adolescent girls in schools when health education campaigns about breast cancer are launched in partnership with schools, parents and community organizations in our study population. As breast cancer screening is likely to increase flow of patients seeking care, any further effort for screening should come along with capacity building of hospitals in detection, treatment and care of people with potential cancers.

The result of this study found that students have a poor knowledge on many breast cancer risk factors, symptoms and curability. There is a need to boost knowledge of students, especially female students. National Health Education, Information and Communication Centre and concerned stakeholders should design awareness programs to sensitize women as well as men, family, and the general community about breast cancer and its early detection measures.

However, the study has few limitations. The instrument used for the assessment of knowledge has not been validated in the Nepalese context, so, room for questioning the construct validity of the tool is present. Besides, all the schools included in this study were public and were drawn from one hilly district of Nepal. This might limit the generalization of our findings to students from private schools and to other districts from the plains. Still, this study will be valuable to frame relevant awareness activities and to guide policy measures considering the context where no literature on breast cancer awareness is available.

## Conclusion

This study demonstrates that students have poor knowledge on many breast cancer risk factors, symptoms and curability. Furthermore, most of the students had misleading information about age, early menarche, delayed menopause and use of OCPs as the risk factors. Many myths regarding curability of breast cancer were prevalent. Breast cancer literacy was significantly better among males in comparison to females. Future studies should particularly aim at identifying measures necessary to address the inadequate knowledge, along with the gaps in school curriculum.

## Additional file

Additional file 1:
**Questionnaire.** (PDF 56 kb)

## Abbreviations

BSE: Breast Self Examination
CBCKT: Comprehensive Breast Cancer Knowledge Test
NPR: Nepalese Rupee
OCP: Oral Contraceptive Pills
