# Supplementary material for: Breast cancer literacy among higher secondary students: results from a cross-sectional study in Western Nepal
Source: BMC Cancer. 2016 Feb 18;16:119. doi: 10.1186/s12885-016-2166-8 (PMC4758038; doi:10.1186/s12885-016-2166-8)
Supplement: Additional file 1: — Questionnaire. (PDF 56 kb) [file 12885_2016_2166_MOESM1_ESM.pdf]

1. Age:………
2. Sex: [ ] Male
   [ ] Female
3. Ethnicity: ……………….
4. Religion: ………………..
5. Grade: …………………..
6. Type of family:…………………...
7. Monthly family income (in NPR):……………….
8. Do you consume any kind of tobacco product?
   [ ] Yes [ ] No
9. Do you consume alcoholic beverages?
   [ ] Yes [ ] No
10. Do you have a family history of cancer?
    [ ] Yes [ ] No
11. Have you heard of Breast Self Examination (BSE)?
    [ ] Yes [ ] No
12. Have you heard of mammography?
    [ ] Yes [ ] No
13. Do you think your school curriculum provides adequate information on cancer?
    [ ] Yes [ ] No
14. From where did you hear about breast cancer?
    [ ] Radio/Television
    [ ] Newspaper
    [ ] Health workers
    [ ] Friends/Relatives
    [ ] Poster/Pamphlets
    [ ] Textbook
    [ ] Teachers
    [ ] Others
15. Which of the following, in your view, are the symptoms of breast cancer?
    [ ] Painless lump in breast
    [ ] Increase in number of breast lumps
    [ ] Pain in breast
    [ ] Discharge of pus from breast
    [ ] Change in size of breast
    [ ] Weight loss
    [ ] Nausea
    [ ] Others
16. Read the following statements on breast cancer. From the options provided with each statement, select the response indicating if you agree, disagree or have no knowledge of the given statement.

I. Early menarche in a woman increases the risk of developing breast cancer.
[ ] Yes [ ] No [ ] Don’t know

II. Women who have delayed menopause are at greater risk of breast cancer.
[ ] Yes [ ] No [ ] Don’t know

III. Use of oral contraceptives increases a woman's risk of developing breast cancer.
[ ] Yes [ ] No [ ] Don’t know

IV. Breastfeeding reduces the risk of developing breast cancer.
[ ] Yes [ ] No [ ] Don’t know

V. Physical activity reduces the risk of developing breast cancer.
[ ] Yes [ ] No [ ] Don’t know

VI. A hard blow to the breast may cause a woman to get breast cancer later in life.
[ ] Yes [ ] No [ ] Don’t know

VII. The constant irritation of a tight bra can, over time, cause breast cancer.
[ ] Yes [ ] No [ ] Don’t know

VIII. In some women, being overweight increases the risk of developing breast cancer.
[ ] Yes [ ] No [ ] Don’t know

IX. A woman who bears her first child before the age of 30 is more likely to develop breast cancer than a woman who bears her first child after the age of 30.
[ ] Yes [ ] No [ ] Don’t know

X. Women with no known risk factors for breast cancer rarely get breast cancer.
[ ] Yes [ ] No [ ] Don’t know

XI. Some types of fibrocystic breast disease (noncancerous breast lumps) increase a woman's risk of breast cancer.
[ ] Yes [ ] No [ ] Don’t know

XII. Breast cancer is more common in 65-year-old women than in 40-year-old women.
[ ] Yes [ ] No [ ] Don’t know

XIII. The most frequently occurring cancer in women is breast cancer.
[ ] Yes [ ] No [ ] Don’t know

XIV. Women over age 70 rarely get breast cancer.
[ ] Yes [ ] No [ ] Don’t know

XV. Most breast lumps are cancerous.
[ ] Yes [ ] No [ ] Don’t know

XVI. For many women, breast cancer can now be successfully treated without breast removal (mastectomy).
[ ] Yes [ ] No [ ] Don’t know

XVII. By the time a cancerous breast lump is painful, it is too late to be successfully treated.
[ ] Yes [ ] No [ ] Don’t know

XVIII. If all lymph glands around the breast and under the arm are not removed, breast cancer cannot be cured.
[ ] Yes [ ] No [ ] Don’t know

XIX. Breast cancer is sometimes treated successfully by removal of the lump (lumpectomy) and radiation therapy.

[ ] Yes [ ] No [ ] Don’t know

XX. Breast cancer is less likely to be cured in women with a family history of breast cancer than in women with no family history of breast cancer.

[ ] Yes [ ] No [ ] Don’t know

XXI. By the time a woman can feel a cancerous breast lump, it is too late to treat it effectively.

[ ] Yes [ ] No [ ] Don’t know

XXII. Even if breast cancer is caught very early, the chances for cure are much better if the whole breast is removed.

[ ] Yes [ ] No [ ] Don’t know

XXIII. Even if detected and treated early, a woman with breast cancer is unlikely to live a normal life span.

[ ] Yes [ ] No [ ] Don’t know
